# Fabrication and Intermolecular Interactions of Silk Fibroin/Hydroxybutyl Chitosan Blended Nanofibers

**DOI:** 10.3390/ijms12042187

**Published:** 2011-03-30

**Authors:** Kui-Hua Zhang, Qiao-Zhen Yu, Xiu-Mei Mo

**Affiliations:** 1 College of Materials and Textile Engineering, Jiaxing University, Zhejiang 314001, China; E-Mail: w2003yqz@126.com; 2 State Key Laboratory for Modification of Chemical Fibers and Polymer Materials, College of Materials Science and Engineering, Donghua University, Shanghai 201620, China

**Keywords:** electrospinning, SF/HBC blends, nanofibrous scaffolds

## Abstract

The native extracellular matrix (ECM) is composed of a cross-linked porous network of multifibril collagens and glycosaminoglycans. Nanofibrous scaffolds of silk fibroin (SF) and hydroxybutyl chitosan (HBC) blends were fabricated using 1,1,1,3,3,3-hexafluoro-2-propanol (HFIP) and trifluoroacetic acid (TFA) as solvents to biomimic the native ECM via electrospinning. Scanning electronic microscope (SEM) showed that relatively uniform nanofibers could be obtained when 12% SF was blended with 6% HBC at the weight ratio of 50:50. Meanwhile, the average nanofibrous diameter increased when the content of HBC in SF/HBC blends was raised from 20% to 100%. Fourier transform infrared spectra (FTIR) and ^13^C nuclear magnetic resonance (NMR) showed SF and HBC molecules existed in hydrogen bonding interactions but HBC did not induce conformation of SF transforming from random coil form to β-sheet structure. X-ray diffraction (XRD) confirmed the different structure of SF/HBC blended nanofibers from both SF and HBC. Thermogravimetry-Differential thermogravimetry (TG-DTG) results demonstrated that the thermal stability of SF/HBC blend nanofibrous scaffolds was improved. The results indicated that the rearrangement of HBC and SF molecular chain formed a new structure due to stronger hydrogen bonding between SF and HBC. These electrospun SF/HBC blended nanofibers may provide an ideal tissue engineering scaffold and wound dressing.

## 1. Introduction

One of the main challenges in tissue engineering (TE) scaffolds is to design and fabricate customizable biodegradable polymeric matrices that mimic the structure and biological functions of the natural extracellular matrix (ECM) [1]. The native ECM is composed of a cross-linked porous network of multifibril collagens with diameters ranging from 50–500 nm and embedded in glycosaminoglycans [2–4]. Silk Fibroin (SF) is a main component of silk worm silk, also an attractive natural fibrous protein for biomedical applications due to its unique properties, including good biocompatibility, good oxygen and water vapor permeability, and biodegradability, low inflammatory response, high tensile strength [5,6]. Silk fibroin has been fabricated as cast films, hydrogels, electrospun nonwoven mats and porous sponges, all of which are widely used for drug delivery, wound dressing and tissue engineering scaffolds [7–10]. Chitosan is a basic natural polysaccharide derived from chitin, which is the second most abundant natural polysaccharide after cellulose. Chitosan has also been widely applied for pharmaceutical and medical fields due to good biocompatibility, biodegradability and various biofunctionalities, including antithrombogenic, hemostaticimmunity enhancing, and wound-healing properties [11–14]. Recently, silk fibroin and chitosan blends have been widely studied as biomaterials in tissue engineering fields to further biomimic components of the native ECM, which have been generally prepared as porous sponges and patches [15,16]. However, biomimic non-woven scaffolds generated by electrospinning have been composed of a large network of interconnected fibers and pores, resembling the topographic features of the ECM [17]. The electrospun nanofibers of silk fibroin and chitosan blends are expected to better biomimic the ECM of native tissues. HBC is fabricated by conjugation of hydroxybutyl (HB) groups to the hydroxyl and amino reactive sites of chitosan. This modification could increase the solubility of chitosan in water or organic solution and electrospinability of chitosan [18], while still remain excellent properties of chitosan [19,20].

The miscibility of its component was critical to the properties of blends. Miscibility in polymer blends is attributed to specific interactions between polymeric components, which are hydrogen bond, ionic and dipole, p-electrons and charge-transfer blend [21]. However, most polymer blends were immiscible due to the absence of molecular interaction. Some reports showed that chitosan could influence physicochemical and biomedical properties of silk fibroin and form a new hydrogen bonding between silk fibroin and chitosan. Meanwhile, chitosan could induce conformation of SF transforming from random coil form to β-sheet structure according to the strong hydrogen bond between CS and SF [22]. On the other hand, more researchers reported that silk fibroin and chitosan blends showed random coil structure of fibroin and transformation to β-sheet structure after treatment by methanol [15,23]. However, these studies were mainly performed in silk fibroin blended casting films or porous sponges. Intermolecular interactions in electrospun silk fibroin-chitosan nanofibers have not been systematically investigated, especially for electrospun SF and HBC blend. In the present study, SF/HBC blended nanofibers were fabricated and intermolecular interaction between SF and HBC were studied, thus developing a new kind of scaffold to biomimic the structure and components for tissue engineering or of functional biomaterials.

## 2. Results and Discussion

### 2.1. Morphology of SF/HBC Nanofibers

**Figure 1.**
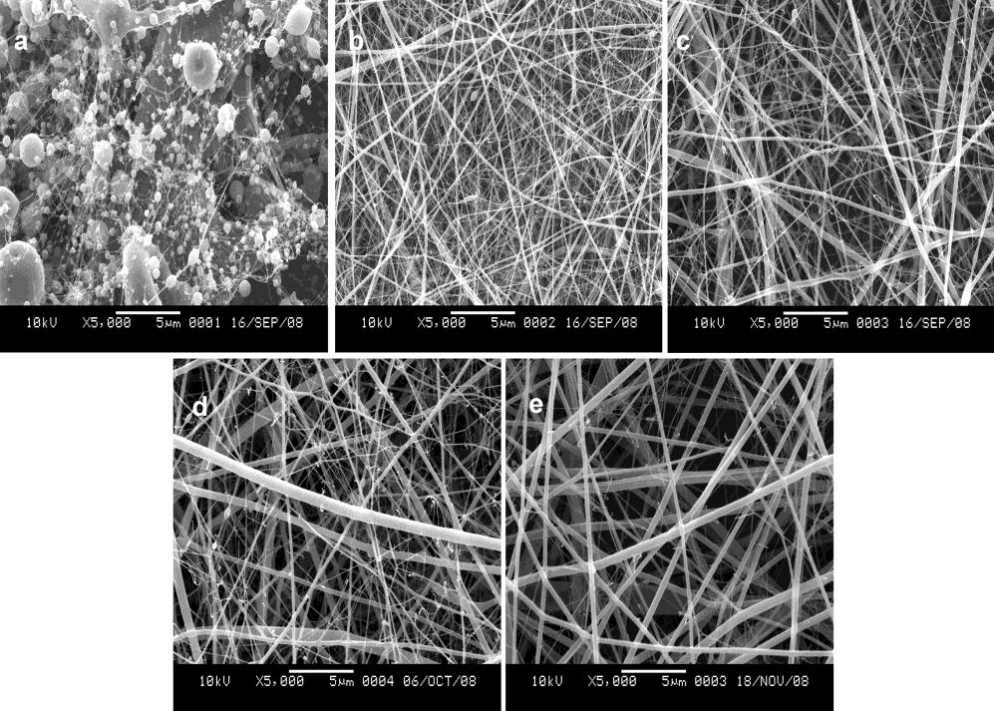
SEM images of electrospun SF/HBC nanofibers with the weight ratio of 50:50 at different concentrations (**a**, 4%SF/4%HBC; **b**, 6%SF/6%HBC; c, 8%SF/8%HBC; **d**, 10%SF/6%HBC; **e**, 12%SF/6%HBC).

In the electrospinning process, HFIP could dissolve SF but not HBC. In order to improve HBC dissolubility, a mixture of HFIP and TFA with volume ratio of 90:10 was selected as an appropriate solvent for electrospinning of HBC. Meanwhile, solution concentration plays a dominant role in determining the fiber morphology, diameter and distribution [24]. The effect of SF/HBC solution concentrations on micrographs was investigated by changing the solution concentrations at the weight ratio of 50:50. SEM morphologies of SF/HBC nanofibers with different concentrations are shown in Figure 1. At a SF/HBC concentration of 4%, several thin nanofibers were initially produced together with large beads; a small quantity of nanofibers with spindle-like beads was observed at 6%. At a SF/HBC concentration of 8%, uniform nanofibers could be obtained. But in the electrospinning process, the needle tube was easily jammed. By adjusting the concentration of SF and HBC until 12% SF was blended with 6% HBC at a ratio of SF to HBC (weight ratio of 50:50), uniform nanofibers are formed. So, we selected a concentration of 12% SF and 6% HBC as following concentration of different ratios of SF/HBC.

**Figure 2.**
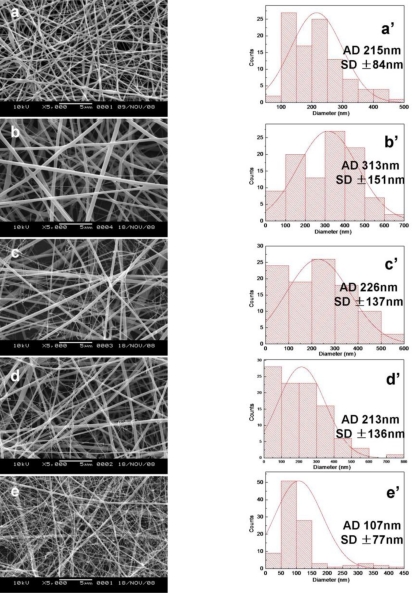
SEM images and diameter distributions at concentration 12%SF/6%HBC with different weight ratios (a,a’, 100:0; b,b’, 80:20; c,c’, 50:50; d,d’, 20: 80; e,e’, 0:100).

The electrospun nanofibers with different blended ratios of SF/HBC from 100:0 to 0:100 were fabricated. The SEM micrographs and diameter distributions of electrospun nanofibers with different weight ratios of SF/HBC from 100:0 to 0:100 are shown in Figure 2. Fiber average diameter of pure SF was 215 ± 84.0 nm. Fiber average diameter increased to 313 ± 151.1 nm after adding 20% HBC, the reason was the increase of solution viscosity. However, fiber average diameter gradually decreased from 313 ± 151.1 nm to 107 ± 77.4 nm on further increasing HBC content in the blend. This effect might be caused by the conductivity increase of the solution with increasing HBC content. Chitosan is a typical cationic polyelectrolyte; more ions were formed in the solution with increasing HBC content. The conductivity of the solution could increase through the addition of ions. On the other hand, the increased charge density will increase elongational forces, which are exerted on the fiber jet to yield a smaller fiber [25].

### 2.2. FTIR Analysis of SF/HBC Nanofibers

**Figure 3.**
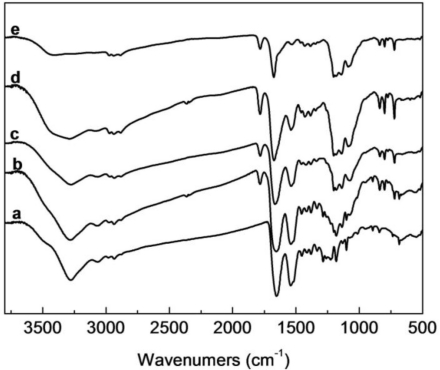
FTIR spectra of electrospun SF/HBC nanofibrous scaffolds with different weight ratios (**a**, 100:0; **b**, 80:20; **c**, 50:50; **d**, 20: 80; **e**, 0:100).

SF has the characteristic absorption bands at 1650–1660 cm^−1^ (amide I), 1535–1545 cm^−1^ (amide II), 1235 cm^−1^ (amide III) and 669 cm^−1^ (amide V), attributed to the SF with random coil conformation, the characteristic bands at 1625–1640 cm^−1^, 1515–1525 cm^−1^, 1265 cm^−1^ and 696 cm^−1^, attributed to the SF with β-sheet conformation [26,27]. FTIR spectra of electrospun SF, HBC, and SF/HBC nanofibers are shown in Figure 3. Electrospun SF, HBC and their blends display characteristic absorption bands between 3278 cm^−1^ and 3405 cm^−1^, which represented the –OH and –NH_2_ group in SF and HBC, the position of characteristic absorption bands were shifted between 3278 cm^−1^and 3405 cm^−1^ in blends with various HBC content. Characteristic absorption bands of –OH and –NH_2_ group have a slight low energy shift due to intermolecular hydrogen bonds. In blends, the absorption bands of amide I, N–H of HBC and amide II of SF, as well as the amide III, both in HBC and SF combined to a single peak which appeared in the middle of the absorption band. Compared to their original position in HBC and SF, the absorption bands were 1653–1676 cm^−1^, 1541–1533 cm^−1^ and 1226−1203 cm^−1^ without remaining primary absorption bands. The results showed intermolecular hydrogen bonding formation of SF and HBC. However, from FTIR spectra analysis, no sign demonstrated transformation of SF from random coil conformation (silk I) to β-sheet structure (silk II) after adding to HBC.

### 2.3. Solid-state ^13^C NMR Analysis of SF/HBC Nanofibers

**Figure 4.**
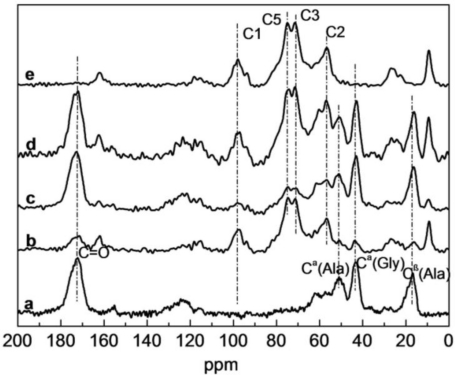
^13^C CP/MAS NMR spectra of SF/HBC nanofibers (**a**, 100:0; **b**, 80:20; **c**, 50:50; **d**, 20: 80; **e**, 0:100).

To further analyze the structure of SF/HBC nanofibers, the ^13^C NMR spectra of pure SF, HBC and SF/HBC nanofibrous scaffolds were performed and shown in Figure 4. In ^13^C NMR spectra of HBC nanofibrous scaffolds, peaks at 97.88, 56.64, 71.41, 82.45, 74.88 and 26.80 ppm were attributed to C1, C2/C6, C3, C4, C5 and methylene /methyl of HBC [28]. In ^13^C NMR spectra of pure SF nanofibrous scaffolds, peaks at 172.2, 60.6, 50.9, 43.3 ppm were attribute to carbonyl carbons of SF, C^β^ of Ser, C^α^ of Ala and C^α^ of Gly [29]. The ^13^C NMR spectra of SF/HBC nanofibrous scaffolds showed characteristic chemical shifts of both SF and HBC. However, after being blended with different ratios, the intensity of some characteristic peaks appeared to change; peak intensities of carbonyl carbons, C^β^ of Ala of SF decreased obviously when blended with 80:20, peak intensities of C1, C2, C3, C5 and methylene/methyl of HBC decreased when blended with 50:50. These peak intensities were not proportional to the content of SF or HBC. In the meanwhile, we found that there were slight changes in the chemical shifts. The results demonstrated that SF and HBC molecules presented H-bond interactions, which led to the change of carbon chemical microenvironment. The chemical shifts of C^β^ of Ala showed HBC did not induce conformation of SF to transform from random coil to β-sheet structure.

### 2.4. X-ray Diffraction Analysis of SF/HBC Nanofibers

**Figure 5.**
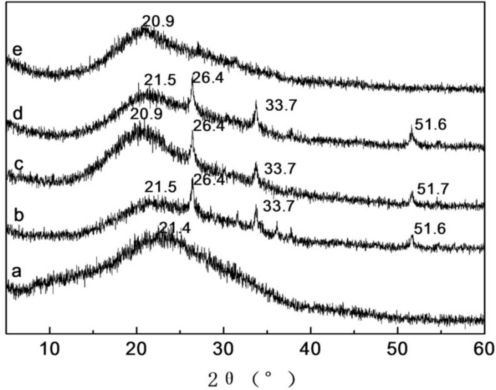
X-ray diffraction spectra of SF/HBC nanofibrous scaffolds with different weight ratios (**a**, 100:0; **b**, 80:20; **c**, 50:50; **d**, 20:80; **e**, 0:100).

X-ray diffraction spectra and crystallinities of pure SF, pure HBC, and SF/HBC nanofibers are shown in Figure 5 and Table 1. HBC nanofibers showed the broad peaks at 20.9, corresponding to amorphous structure of HBC. HBC is fabricated by conjugation of hydroxybutyl (HB) groups to the hydroxyl and amino reactive sites of chitosan. The hydroxylbutyl groups in side chain destroyed the original hydrogen bonding interaction between the chitosan molecules. Pure SF nanofibers showed only a broad peak centered at 21.4°, which was the characteristic peak of silk I [30]. Thus pure SF existed mainly in random coil structure. SF/HBC nanofibers showed three new peaks at 26.4° (*d* = 3.37 Å), 33.7°(*d* = 2.66 Å), and 51.8°(*d* = 1.77 Å) and strong intensities without either pure SF or HBC nanofibers. Furthermore, crystallinities of SF/HBC nanofibers at different weight ratios increased in comparison with pure SF or HBC nanofibers. However, FTIR showed HBC did not induce conformation of SF transforming from random coil form to β-sheet structure. The results indicated rearrangement of molecular chain HBC and SF could form a new structure due to strong interaction between SF and HBC. The molecular interactions between SF and HBC may be produced by hydrogen bonds formation. The –OH groups, C═O groups and -NH_2_ groups in SF are capable of forming hydrogen bonds with –OH and –NH_2_ groups in HBC. From X-ray diffraction analysis, SF and HBC mainly existed in amorphous structure. The original chitosan molecules interactions were weakened due to the existence of hydroxyl butyl groups. Hence, SF and HBC molecules are prone to form hydrogen bond interactions when SF is blended with HBC leading to a new structure.

### 2.5. Thermal Analysis of SF/HBC Nanofibers

**Figure 6.**
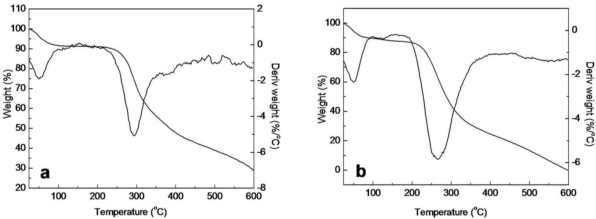
TG-DTG curves of raw SF and HBC (**a**, SF; **b**, HBC).

**Figure 7.**
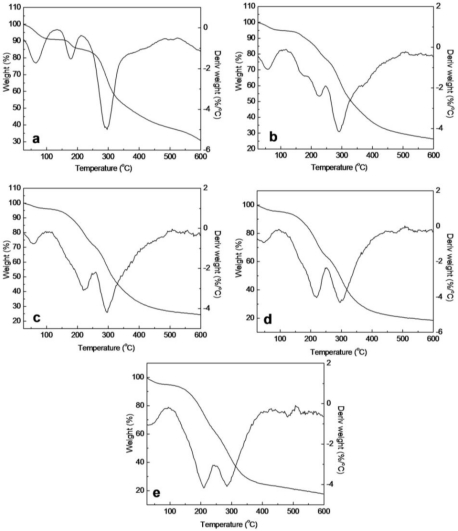
TG-DTG curves of electrospun SF/HBC nanofibers (**a**, 100:0; **b**, 80:20; **c**, 50:50; **d**, 20:80; **e**, 0:100).

In order to investigate the thermal behavior of decomposition in detail, TG differential thermogravimetric (DTG) curves of raw SF, elelctrospun SF, electrospun SF/HBC nanofibers, electrospun HBC and raw HBC, were obtained and shown in Figures 6 and 7. The thermal decomposition temperature and mass loss in several stages are shown in Table 2. The mass loss of raw SF at the first stage (24–215 °C) was connected with the evaporation of water (9.05%). The mass loss rate increased and attained its maximum at 289.5 °C, which resulted from the disintegration of intermolecular interaction and the partial breakage of the molecular structure (61.72%) [31]. However, electrospun SF nanofibrous showed a new mass loss stage (147–210 °C) and attained its maximum at 177.2 °C in comparison with raw SF; this was possibly caused by interactions between SF and solvent(HFIP) leading to degradation of H-bonds in crystal sites. The mass loss of raw HBC at the first stage (25–185 °C) is attributed to the evaporation of water (12.74%). The mass loss rate increased and attained its maximum at 263.5 °C, ascribed to a blend process including dehydration of the saccharide rings and decomposition of the hydroxybutyl groups units. Meanwhile, the onset thermal decomposition temperature of electrospun HBC nanofibrous scaffold advanced to 122 °C and was divided into two decomposition peaks, attaining a maximum at 210.7 °C and 284.6 °C, respectively. This can be explained by the fact that TFA of HFTP/HFA blended solvent forms salts with the amino groups of HBC and this salt formation destroyed the rigid H-bond interaction between the HBC molecules leading to lower thermal stability than raw HBC.

For electrospun SF/HBC nanofibers, the maximum decomposition temperature improved 9–13 °C at the stage of 122–250 °C and 7–16 °C at the stage of 250–600 °C in comparison with electrospun HBC nanofibrous scaffold. Meanwhile, the mass loss peaks of SF disappeared at the stage of 147–210 °C compared with pure SF. The results indicated that intermolecular hydrogen bonds gave rise to more thermal stability than single component.

## 3. Experimental Section

### 3.1. Materials

Cocoons of Bombyx mori silkworm were kindly supplied by Jiaxing Silk Co. Ltd (China). hydroxybutyl chitosan was kindly provided by Shanghai Qisheng biological agents Co. Ltd (China). Two kinds of solvents, 1,1,1,3,3,3-hexafluoroisopropanol (HFIP) from Fluorochem Ltd. (U.K.) and trifluoroacetic acid (TFA) from Sinopharm Chemical Reagent Co., Ltd. (China).

### 3.2. Preparation of Regenerated SF

Raw silk was degummed three times with 0.5% (w/w) Na_2_CO_3_ solution at 100 °C for 30 min each and then washed with distilled water. Degummed silk was disolved in a ternary solvent system of CaCl_2_/H_2_O/EtOH solution (1/8/2 in mole ratio) for 1h at 70 °C. After dialysis with cellulose tubular membrane (250-7u; Sigma) in distilled water for 3 days at room temperature, the SF solution was filtered and lyophilized to obtain the regenerated SF sponges.

### 3.3. Electrospinning of SF/HBC Nanofibers

The SF was dissolved in HFIP and HBC was dissolved in HFIP/TFA mixture (v/v 90:10) for various concentrations, respectively. When they were prepared already, the two solutions were blended at different weight ratios with sufficient stirring at room temperature before electrospinning. All of the electrospinning procedures were carried out at the environmental conditions with the temperature of 25–30 °C and the relative humidity of 30–40%. The solutions were placed into a 2.5 mL plastic syringe with a blunt-ended needle with an inner diameter of 0.21 mm. The needle was located at a distance of 200 mm from the grounded aluminum foil collector. A syringe pump (789100C, cole-pamer, USA) was employed to feed solutions to the needle tip at a feed rate of 0.5–1.0 mL/h. A high electrospinning voltage was applied between the needle and ground collector using a high voltage power supply (BGG6-358, BMEICO.LTD, China). The applied voltage was 20 KV. The electric field generated by the surface charge caused the solution drop at the tip of the needle to distort into a Taylor cone. The electrospun SF/HBC were dried in a vacuum (vacuum degree is −0.1 MPa) oven for more than 15 days at room temperature.

### 3.4. Characterization

The morphology and diameter of the electrospun fibers was observed with a scanning electronic microscope (SEM) (JSM-5600, Japan) at an accelerated voltage of 10KV. The diameter range of the fabricated nanofibers(×10,000) was measured based on the SEM images using an image visualization software Image J 1.34s (National Institutes of Health, USA) and calculated by selecting 100 fibers randomly observed on the SEM images.

Fourier transform infrared spectra (FTIR) were obtained on AVATAR 380 FTIR instrument (Thermo Electron, Waltham, MA, USA). All spectra were recorded by an absorption mode in the wave length range of 4000–500 cm^−1^.

The ^13^C CP-MAS nuclear magnetic resonance spectra (NMR) of the electrospun scaffolds were obtained on NMR spectrometer (Bruker AV400, Switzerland) with a ^13^C resonance frequency of 100 MHz, contact time of 1.0 ms, pulse delay time of 4.0 s.

Wide-angle X-ray diffraction (WAXD) curves were obtained on an X-ray diffractometer (RigaKu, Japan) within the scanning region of 2 θ (5°–50°), with Cu Ka radiation (λ = 1.5418 A°) at 40 kV and 40 mA.

Thermogravimetry (TG) was carried out in air with the use of a TG 209F1 thermogravimetric system (Netzsch Germany) within the temperature range from room temperature to 600 °C at a heating rate of 10 °C min^−1^.

## 4. Conclusions

The SF/HBC nanofibrous scaffolds were fabricated via electrospinning. Through adjusting the concentrations of SF and HBC, relative uniform nanofibers could be obtained when 12% SF was blended with 6% HBC at the weight ratio of 50:50 and nanofiber diameter decreased from 313 ± 151.1 nm to 107 ± 77.4 nm on increasing the content of HBC from 20% to 100%. XRD and TG-DTG results demonstrated SF/HBC nanofibrous scaffolds formed a new structure and thermal stability improved. The results demonstrated SF might form different types of hydrogen bonds with HBC, between –OH groups, C═O groups and –NH_2_ groups in SF and –OH and –NH_2_ groups in HBC. The intermolecular hydrogen bonds between SF and HBC were superior to hydrogen bonds between molecules of the same polymer. This gave rise to the rearrangement of molecular chain HBC and SF to form a new structure. Our ongoing studies will focus on the application of the electrospun SF/HBC nanofibers in tissue engineering and in wound dressing.

## Figures and Tables

**Table 1.** Crystallinities of SF/HBC nanofibrous scaffolds.

**Content of HBC**	**Crystallinities (%)**	**Content of HBC**	**Crystallinities (%)**
0	16.9	80	24.9
20	25.0	100	14.6
50	26.1		

**Table 2.** The thermal decomposition temperature and mass loss in several stages.

	**Raw SF**	**Electrospun SF**	**Electrospun SF/HBC blends**			**Electrospun HBC**	**Raw HBC**
			**80:20**	**50:50**	**20:80**		
First stage (°C)	24–215	24–147	25–122	27–122	25–122	25–122	25–185
T_max1_ (°C)	48.9	65.5	54.1	53.0	50.2	41.1	47.9
Loss mass (%)	9.05	9.15	5.36	4.36	5.53	6.05	12.74
Second stage (°C)	215–600	147–210	122–250	122–251	122–250	122–250	185–600
T_max2_ (°C)	289.5	177.2	223.5	221.3	219.8	210.7	263.3
Loss mass (%)	61.72	5.54	18.08	23.88	30.60	44.43	87.26
Third stage (°C)	—	210–600	250–600	251–600	250–600	250–600	—
T_max3_ (°C)	—	293.7	291.4	293.3	300.4	284.6	—
Loss mass (%)	—	54.34	50.14	47.32	45.43	42.53	—
Residues (%)	29.2	30.97	26.42	24.44	18.44	17.99	0

## References

[1] Langer R, Vacanti JP (1993). Tissue engineering. Science.

[2] Xu CY, Inai R, Kotaki M, Ramakrishna S (2004). Aligned biodegradable nanofibrous structure: a potential scaffold for blood vessel engineering. Biomaterials.

[3] Min BM, Lee G, Kim SH, Nam YS, Lee TS, Park WH (2004). Electrospinning of silk fibroin nanofibers and its effect on the adhesion and spreading of normal human keratinocytes and fibroblasts *in vitro*. Biomaterials.

[4] Wang M, Jin HJ, David L, Rutledge GC (2004). Mechanical properties of electrospun silk fibers. Macromolecules.

[5] Santin M, Motta A, Freddi G, Cannas M (1999). *In vitro* evaluation of the inflammatory potential of the silk fibroin. J. Biomed. Mater. Res.

[6] Horan RL, Antle K, Collette AL, Huang YZ, Huang J, Moreau JE, Volloch V, Kaplan DL, Altman GH (2005). *In vitro* degradation of silk fibroin. Biomaterials.

[7] Vepari C, Kaplan DL (2007). Silk as a biomaterial. Prog. Polym. Sci.

[8] Numata K, Subramanian B, Currie HA, Kaplan DL (2009). Bioengineered silk protein-based gene delivery systems. Biomaterials.

[9] Jiang X, Zhao J, Wang S, Sun X, Zhang X, Chen J, Kaplan DL, Zhang Z (2009). Mandibular repair in rats with premineralized silk scaffolds and BMP-2-modified bMSCs. Biomaterials.

[10] Mandal BB, Kundu SC (2009). Cell proliferation and migration in silk fibroin 3D scaffolds. Biomaterials.

[11] Strand SP, Lelu S, Reitan NK, De Lange Davies C, Artursson P, Varum KM (2010). Molecular design of chitosan gene delivery systems with an optimized balance between polyplex stability and polyplex unpacking. Biomaterials.

[12] Chen MC, Wong HS, Lin KJ, Chen HL, Wey SP, Sonaje K, Lin YH, Chu CY, Sung HW (2009). The characteristics, biodistribution and bioavailability of a chitosan-based nanoparticulate system for the oral delivery of heparin. Biomaterials.

[13] Tchemtchoua VT, Atanasova G, Aqil A, Maquet V, Jerôme C, Poumay Y, Colige A (2009). Development of a procedure to simultaneously isolate RNA, DNA, and proteins from characterizing cells invading or cultured on chitosan scaffolds. Anal. Biochem.

[14] Thanou BI, Florea M, Geldof HE, Junginger HE, Borchard G (2002). Quaternized chitosan oligomers as novel gene delivery vectors in epithelial cell lines. Biomaterials.

[15] She ZD, Jin CR, Huang Z, Zhang BF, Feng QL, Xu YX (2008). Silk fibroin/chitosan scaffold: Preparation, characterization and culture with HepG2 cell. J. Mater. Sci. Mater. Med.

[16] Yang MC, Wang SS, Chou NK, Chi NH, Huang YY, Chang YL, Shieh MJ, Chung ZW (2009). The cardiomyogenic differentiation of rat mesenchymal stem cells on silk fibroin-polysaccharide cardiac patches *in vitro*. Biomaterials.

[17] Li WJ, Laurencin CT, Caterson EJ, Tuan RS, Ko FK (2002). Electrospun nanofibrous structure: A novel scaffold for tissue engineering. J. Biomed. Mater. Res.

[18] Dang JM, Leong KW (2007). Myogenic induction of aligned mesenchymal stem cell sheets by culture on thermally responsive electrospun nanofibers. Adv. Mater.

[19] Chen BY, Dang JY, Tan TL, Fang N, Chen WN, Leong KW, Chan V (2007). Dynamics of smooth muscle cell deadhesion from thermosensitivehydroxybutyl chitosan. Biomaterials.

[20] Dang JM, Sun DD, Shin-Ya Y, Sieber AN, Kostuik JP, Leong KW (2006). Temperature-responsive hydroxybutyl chitosan for the culture of mesenchymal stem cells and intervertebral disk cells. Biomaterials.

[21] Sionkowsk A, Wisniewski M, Skopinsk J, Kennedy CJ, Wess TJ (2004). Molecular interactions in collagen and chitosan blends. Biomaterials.

[22] Chen X, Li WJ, Yu TY (1997). Conformation transition of silk fibroin induced by blending chitosan. J. Appl. Polym. Sci. Part B: Polym. Phys.

[23] Kweon A, Ha HC, Um IC, Park YH (2001). Physical properties of silk fibroin/chitosan blend films. J. Appl. Polym. Sci.

[24] Huang CB, Chen SL, Lai CL (2006). Electrospun polymer nanofibres with small diameters. Nanotechnology.

[25] Zong XH, Kim K, Fang DF, Ran SF, Hsiao BS, Chu B (2002). Structure and process relationship of electrospun bioabsorbable nanofiber membranes. Polymer.

[26] Magoshi J, Mizuide M, Magoshi Y (1979). Physical properties and structure of silk—VI conformational changes in silk fibroin induced by immersion in water at 2 to 130 °C. J. Polym. Sci. Polym. Phys. Edit.

[27] Chen X, Shao ZZ, Marinkovic NS, Miller LM, Zhou P, Chance MR (2001). Conformation transition kinetics of regenerated *Bombyx mori* silk fibroin membrane monitored by time-resolved FTIR spectroscopy. Biophys. Chem.

[28] Fernández Cervera M, Heinämäki J, Räsänen M, Maunu SL, Karjalainen M, Nieto Acosta OM, Iraizoz Colartea A, Yliruusi J (2004). Solid-state characterization of chitosans derived from lobster chitin. Carbon. Polym.

[29] Asakura T, Iwadate M, Demura M, Williamson MP (1999). Structural analysis of silk with ^13^C NMR chemical shift contour plots. Int. J. Biol. Macromol.

[30] Asakura AK, Tabeta R, Saito H (1985). Conformational characterization of Bombyx mori silk fibroin in the solid state by high-frequency carbon-13 cross polarization-magic angle spinning NMR, X-ray diffraction, and infrared spectroscopy. Macromolecules.

[31] Du CH, Zhu BK, Chen JY, Xu YY (2006). Metal ion permeation properties of silk fibroin/chitosan blend membranes. Polym. Int.

